# The effects of daily autobiographical memory training on memory bias, mood and stress resilience in dysphoric individuals

**DOI:** 10.1038/s41598-022-25379-9

**Published:** 2022-12-03

**Authors:** Leonore Bovy, Nessa Ikani, Livia N. M. van de Kraats, Martin Dresler, Indira Tendolkar, Janna N. Vrijsen

**Affiliations:** 1 Donders Institute of Cognition and Behaviour, Radboud University Medical Center, Centre for Cognitive Neuroimaging, Nijmegen, The Netherlands; 2 Department of Psychiatry, Donders Institute for Brain, Cognition and Behaviour, Radboud University Medical Center, Nijmegen, The Netherlands; 3 Behavioural Science Institute, Radboud University, Nijmegen, The Netherlands; 4 Depression Expertise Centre, Pro Persona Mental Health Care, Nijmegen, The Netherlands

**Keywords:** Psychology, Human behaviour, Depression

## Abstract

Negative memory bias refers to the enhanced recall of negative memories and is a prominent cognitive factor causing and maintaining depression. Surprisingly few studies modify this negative recall. The current study used a smartphone-based autobiographical memory training to increase positive memory recall and thereby alter negative memory bias. A total of 96 dysphoric (≥ 13 BDI-II) participants were randomly allocated to a positive, sham or no-training condition, conducted over a period of 6 days. Positive memory bias (i.e., recalled event evaluation) significantly increased from pre- to post-training after positive and sham intervention, suggesting an unspecific training effect. No transfer to memory specificity, implicit memory bias or depressive symptoms was found, nor was the training effect modulated by pre-existing level of positive memory bias. A post-hoc follow-up measurement during the initial COVID-19 crisis revealed that subjects who benefitted most from either of the trainings maintained their stress levels better during a natural stressful period, compared to those who responded least to the training. Future studies should carefully consider the impact of sham training design. Moreover, it is important to examine transfer effects of bias training as practice in daily life.

## Introduction

Depressed individuals show preferential processing of negative information^1,2^. Mood-congruent emotional information is more likely to be attended to and processed than mood-incongruent information, through a spreading of activation of related nodes within an associative network^3^. The activation of a negative node (e.g., by encountering something negative) leads to the activation of a negative or even depressive network, which in turn biases the processing of new information, thus increasing or perpetuating an already existing negative bias. The cognitive theory of depression^4^ emphasizes the role of negative processing biases in the onset, maintenance, and recurrence of depression. Negative memory bias is the tendency to remember negative information better than neutral or positive^5,6^ and is of particular relevance in depression. Also reduced autobiographical memory specificity (i.e., the lack of detail in memory recollection) is a known risk factor for depression^7^.

Several studies have shown that self-relevance is an important moderator for biased recall in analyses that compared clinically-depressed to nondepressed groups^5,8^. Moreover, depressed patients tend to recall generic autobiographical experiences more than memory-specific ones^9^ and evaluate past and future autobiographical events as more negative and less positive^10^, which has been related to rumination^11^. In addition, mood-congruent biases have been shown in implicit non-autobiographical memory recall^5^. Within the well-established Deese-Roediger-McDermott (DRM) false memory paradigm^12^, Major Depressive Disorder patients demonstrate a greater false memory for negative critical lures, in both free recall^13^ and recognition^14^, but the relation with biased autobiographical experience is unclear.

Cognitive bias modification (CBM) methods, aimed at alleviating anxiety or depression, mostly targeted and attenuated attention or interpretation biases—showing mixed results, most probably caused by the different types of methodology used to modify these biases and the different ways in which they may operate in anxiety and depression^15,16^. The manipulation of emotional memory bias, however, received little attention. Early CBM trainings mainly focused on training forgetting of negative information^17^. Yet, manipulation of the specificity and flexibility, but not the selectivity, of memory, has received more extensive attention yielding promising clinically relevant results (e.g., reducing depressive symptomatology)^18–20^. It is important to take into account though, that repeated retrieval is a powerful learning strategy that can promote transfer of the learned information to other contexts^21,22^. Indeed, experimental lab trainings using positive memory recall in healthy samples^23–25^ or in vulnerable dysphoric and high-ruminating samples^26^ show initial success in changing bias. Moreover, in one study^26^, a transfer towards a more positive autobiographical memory bias was found which was dependent on a pre-existing self-reference-related positive bias. This suggests that participants who already showed a more positive bias before training benefit the most of training, as expressed in a more positive autobiographical memory bias.

Although (the modification of) memory bias has predominantly been investigated in the lab, mobile health apps could be utilized as effective and more ecologically valid modification and sampling tools to assess subjective experiences throughout daily life^27^. In light of these advantages, a recent study^28^ developed a new smartphone-based autobiographical memory training to study the effects of training on memory bias and transfer to mood symptoms in a healthy sample, using the experience sampling method (ESM). ESM is a promising technique to assess dynamic aspects of cognition (e.g., memory bias or mood) multiple times throughout the day^27^. Administered through a smartphone app, training can take place in the participant’s natural environment, thereby increasing the possibility of transferring training effects to daily life cognitive processing. In our previous pilot study in an unselected sample^28^, the positive training led to increased positive memory bias post-training, but this effect did not differ significantly from the neutral or negative training conditions. In addition, the positive training yielded a higher proportion of recall of positive autobiographical events. Although training effects were positive, no clinically relevant outcomes were found (i.e., transfer to self-referential explicit memory, autobiographical memory or depressive symptoms). This may have been related to floor-level performance of the unselected healthy sample or an insufficient training dosage (3 days). Moreover, long-term effects could not be investigated due to the lack of a follow-up measurement.

The current study follows up on our earlier pilot study^28^, by using a slightly adapted version of the training in a clinically relevant, vulnerable sample of dysphoric students instead of an unselected sample. Training length was extended from 3 to 6 days to increase the therapeutic dose of the training. The main outcome measurements were supplemented by depression and rumination questionnaires and memory bias measurements. Besides transfer to self-relevant explicit memory (which is central to depression^29^), we also assessed the width and specify of transfer by including tasks assessing different aspects of depressotypic memory. Hence, we measured autobiographical memory specificity and memory intrusions within a false memory task^13,30^. Moreover, the current sham training was designed for participants to describe and evaluate their environmental context, reflecting a more factual experience. A no-training control group was included to measure the natural course of depressive symptoms and to probe potential non-specific training effects. A negative training was omitted as this was regarded as not ethical in an already vulnerable dysphoric sample. The effect of training on overall memory bias and mood was measured using ESM. Lastly, a follow-up session was added post-hoc to explore long-term training effects and potential resiliency during a generally stressful period—the global COVID-19 outbreak (May 2020).

Overall, the positive training was hypothesized to show an increase in positive recall from pre- to post-measurement. In addition, the transfer effect of the positive training on memory bias measurements, depressive symptoms and rumination was hypothesized to be dependent on baseline level of memory bias^26^. We also hypothesized that a neutral sham training would not change biased recall of positive and negative memories. Lastly, we expected the positive training to show the strongest decrease in self-reported depressive symptoms.

## Methods

### Participants

A total of 96 dysphoric participants (mean age 24.01 ± 6.71; range 18–56 years; 80 females; 66 Dutch/30 German) were recruited via the Radboud University (The Netherlands) online participant recruitment system (SONA) in return for course credit or monetary compensation. Participants were pre-screened for elevated scores ($\ge$ 13) of the Beck Depression Inventory-II (BDI-II^31^) and fluency in Dutch or German. Participants were randomly assigned to the positive training (*n* = 32), sham training (*n* = 32) or no-training (control; *n* = 32) condition. A randomization list was created based a random number generator before the start of the study and monitored by hand. Upon informed consent completion, each participant was randomized based on the list in the order they entered the study. The study was double-blind as both participants and researchers were unaware of the participant’s condition allocation. One participant in the no-training condition dropped out after the baseline measurement. Moreover, two participants were exluded due to technical failure of prompts delivery during the training, resulting in 31 participants per condition. All assessment and training material was available in Dutch and German.

The study was conducted in accordance with the Declaration of Helsinki and approved by the Radboud University Social Sciences ethical committee (Protocol ID: ECSW-2018-047) and informed consent was obtained from all participants. In addition, the study was pre-registered at AsPredicted under ‘MEDAL3’ (registration number: 24996). We planned to recruit 150 participants, based on calculating a medium effect size with a power of 0.8, however, lab-based data collection was halted earlier due to the COVID-19 outbreak (March 2020).

### Experimental procedure

Each condition included a lab-based baseline session and a post-training session (6 days after training). At baseline, participants filled out questionnaires (demographic information, BDI-II, Depression Anxiety Stress Scales (DASS), Ruminative Response Scales (RRS), Positive Mental Health Scale (PMHS)), completed the Self-Referent Encoding Task (SRET), measuring memory bias^32^ and an emotional false memory task^12^. At the post-training session, participants again filled out all questionnaires (BDI-II, DASS, RRS, PMHS), repeated the emotional false memory task and completed an autobiographic memory task (AMT)^33^. During the early stages of the COVID pandemic, all participants were invited per email to participate in the online follow-up phase. A total of *n* = 34 (positive training = 12, sham training = 15, no-training = 7) completed a follow-up online questionnaire, on average 54.7 weeks after the original study (range 9.2–98.9; May 2020).

### Experience sampling method measurements

CBM was conducted using ESM^27^ with a smartphone app (created with MovisensXS application; xs.movisens.com), in the positive and sham training condition. The no-training condition did not receive a smartphone app. Each of the six training days included eight prompts delivered between 08:00 and 22:00. The first prompt was activated by participants themselves via a button press; which was available between 08:00 and 09:45 h. Subsequent prompts were sent at random 1 h–45 min intervals (see Figure S1 for an overview).

The first and last training day included only three positive or sham training prompts because the rest were memory bias or mood assessment prompts. All other days included five positive or sham training prompts per day, given that the other three were memory bias or mood assessment prompts. During the positive training, participants were asked to recall the most pleasant event that happened since the last prompt or since awakening that morning, describe it in minimally five keywords, and evaluate it on a continuous scale ranging from “extremely unpleasant” (− 50) to “extremely pleasant” (+ 50). In the sham training, participants were asked to limit the description to their location and company (i.e., contextual environment) using five keywords, and evaluate it on the same scale as described above. Keywords were manually analyzed post-training to check for non-adherence to the training protocol (i.e., systematically not responding to questions (e.g., empty form/typing random letters) or repeatedly describing the same event.

Memory bias was assessed three times on the first training day (i.e., first prompts of the day before training onset), at the end of the last day, and once per day for the remainder of the training (timing varied across days; Figure S1). The assessment items requested participants to recall the most important event since the last prompt or since waking up that morning and to evaluate it on a scale from “extremely unpleasant” (− 50) to “extremely pleasant” (+ 50). Average of the first and last three measurements and the change in these aggregated scores per condition were used as a manipulation check. In line with^28^, each rating was additionally dichotomized as positive (> 0 into 1) or negative (< 0 into 0). A sum score was calculated per timepoint: a score of “3” indicated overall positive bias, “2” a moderate positive bias, “1” a moderate negative bias, and “0” a negative bias.

Four questions on current mood, stating “I feel happy/relaxed/sad/stressed”, were evaluated on a scale from “not at all” (0) to “very much” (100), reflecting the central Valence (positivity/negativity) × Arousal (degree of mental alertness or activation) axes^34–36^.

### Other tasks

For more details on the SRET, AMT, emotional false memory task and questionnaires, please refer to the Supplementary Materials.

### Statistical analyses

All analyses were conducted in the R studio (version 4.0.0.^37^). Differences between the experimental conditions (positive, sham, no-training) across time (baseline, post-training) were analyzed using repeated measures analysis of variance (ANOVA) tests. Analyses related to memory bias were performed between the positive and sham training. Simple main effects were explored in Bonferroni-corrected post-hoc tests. Linear mixed-effects models were used to control for multiple measurements of different participants across multiple time points, using the *lmer* function from the lme4 R package^38^. P values were determined using Type 3 Likelihood Ratio tests using the mixed function of the *afex* package^39^. Differences in frequencies were tested with chi-square tests. The alpha level was set at 0.05.

## Results

### Baseline characteristics

The groups did not differ in average age or any of the baseline questionnaires (all p > 0.1; see Table 1). Due to a programming error, we accidentally applied a BDI-II cut-off ≥ 13 and not ≥ 14 (the cutoff value representing mild depression). However, the sample demonstrated light depressive symptoms (BDI-II score: 18 ± 0.855; range 0–48) where 71% of participants scored ≥ 14. At baseline, prior memory bias was assessed using the SRET. After exclusion of one participant (due to total memory failure), overall positive recall bias was high (0.74 $\pm$ 0.23), while conditions did not differ significantly (*F*(2, 92) = 2.497, *p* = 0.088, ${\eta }^{2}$ = 0.051).

**Table 1:** Baseline characteristics.

	Positive training *n* = 32	Sham training *n* = 32	No training *n* = 32	Group comparisons
Gender (F/M)	30/2	24/8	26/6	χ^2^ (2) = 4.2, *p* = 0.123
Language (D/G)	23/9	21/11	22/10	χ^2^ (2) = 0.29, *p* = 0.865
Age	23.3 ± 1.06	23.2 ± 0.807	25.5 ± 1.56	*F*(2, 93) = 1.19, *p* = 0.309
BDI-II	17.9 ± 1.73	16.5 ± 1.17	19.5 ± 1.49	*F*(2, 93) = 1.01, *p* = 0.37
RRS	48.5 ± 1.98	52 ± 2.06	54 ± 2	*F*(2, 93) = 1.93, *p* = 0.151
PMHS	24.4 ± 0.983	24.5 ± 0.797	23 ± 0.836	*F*(2, 93) = 0.96, *p* = 0.386
DASS depression	13.6 ± 1.77	10.9 ± 1.37	12 ± 1.46	*F*(2, 93) = 0.80, *p* = 0.451
DASS anxiety	8.88 ± 1.33	9.69 ± 1.36	9.94 ± 1.47	*F*(2, 93) = 0.50, *p* = 0.61
DASS stress	14.3 ± 1.34	14.4 ± 1.52	16.1 ± 1.51	*F*(2, 93) = 0.16, *p* = 0.852
SRET	0.79 ± 0.23	0.76 ± 0.23	0.67 ± 0.22	*F*(2, 93) = 2.497, *p* = 0.088

Counts, means, standard errors (SE) and group comparisons of baseline measurements.

*F* female, *M* male, *D* Dutch, *G* German, *BDI*-*II* Beck’s Depression Inventory-Revised Version, *RRS* Ruminative Response Scale, *PMHS* Positive Mental Health Scale, *DASS* Depression Anxiety Stress Scales, *SRET* Self-Referent Encoding Task.

Participants spent on average 27.56 (± 36.72, range 0–559) seconds per prompt and used the app on average 7.2 (± 0.65, range 1.27–22.93) minutes per day. Compliance rate was high (~ 96%) and did not differ between conditons (*t*(59) = 0.46, *p* = 0.65).

### Memory bias (ESM)

#### Training effects on ESM memory bias

**Figure 1:**
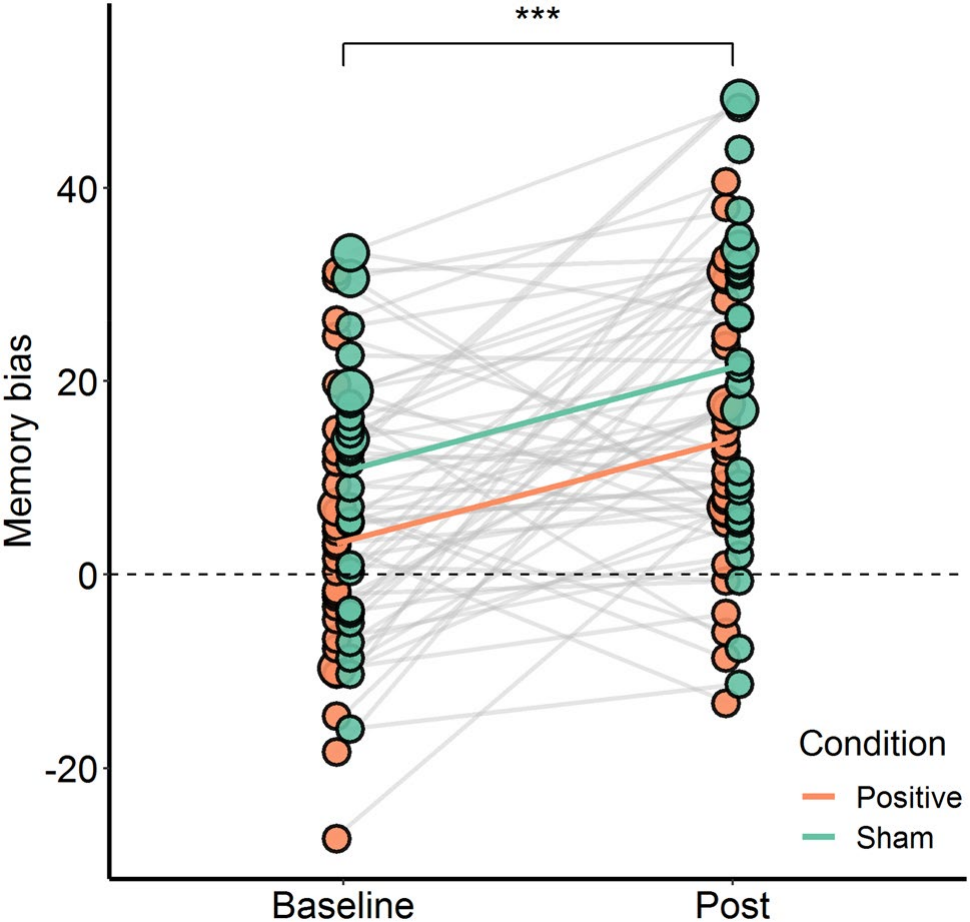
Memory bias scores before and after training. A higher positive value reflects a stronger positive bias. Dots represent the individual participants, where larger dots represent multiple participants with the same value. Individual slopes between baseline and post measurement are depicted as grey lines, whereas condition averages are represented as colored lines. The plot depicts a significant main effect of time and main effect of group. ***p < 0.001.

To explore the influence of the training on overall memory bias, the average recalled event evaluation of the three memory bias measurements at the start and the end of the study were investigated, but no significant Time × Condition interaction was found (*F*(1, 60) = 0.003, *p* = 0.954, ${\eta }^{2}$ = 0.00002). A main effect of time was found, (*F*(1, 60) = 20.417, *p* < 0.001, ${\eta }^{2}$ = 0.122), indicating an overall increase towards positive bias in both conditions ($\Delta$*M* = 10.64). A main effect of condition was found, (*F*(1, 60) = 7.375, *p* = 0.009, ${\eta }^{2}$ = 0.068). Post-hoc pairwise comparisons revealed that the sham condition on average scored a more positive bias than the positive condition ($\Delta$*M* = 5.14, *p* = 0.006, see Fig. 1). Lastly, there were no modulating effects of prior positive bias and BDI-II score (see Supplementary Materials for more details).

Given the difference in positive memory bias between the positive and sham condition, we explored the difference between the conditions on the scores reported during the training (i.e., when either recalling a positive event or rating the contextual environment). After averaging all training recall scores per participant, conditions statistically differed from each other (*F*(1, 60) = 4.475, *p* = *0.*039, *η*^2^ = *0.*069), where the sham condition rated their contextual environment higher (*M* = 20.9) compared to the positive training condition (*M* = 14.8).

#### Training effects on ESM memory bias proportional scores

**Figure 2:**
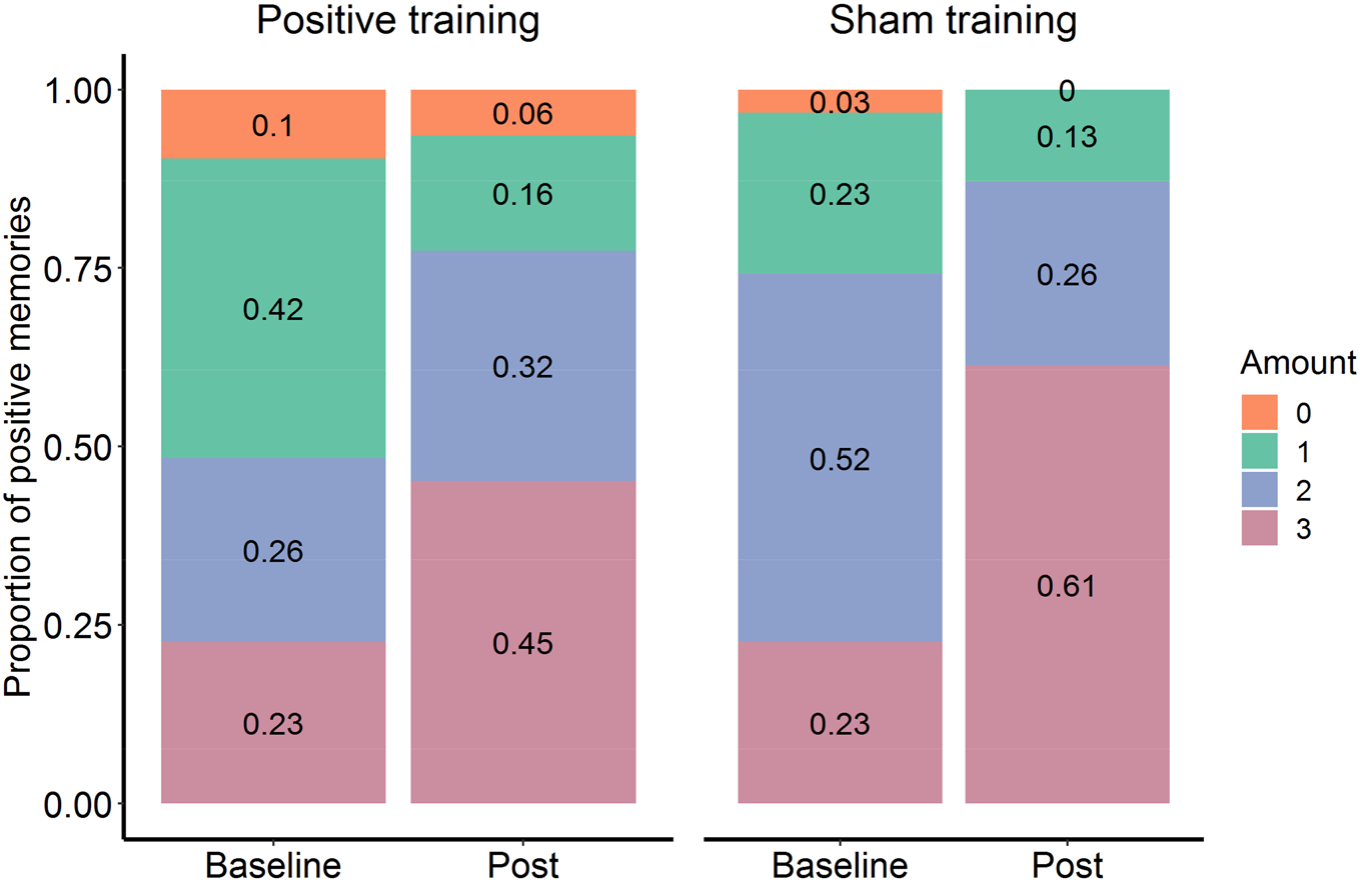
Proportional positive memory before and after training. Sum scores on the amount of positive memories per condition over time, depicted as proportions.

After creating a sum score of overall positive memories per participants, a chi-square test for the positive training condition was performed, revealing no significant Valence (4 levels) × Time (2 levels) interaction (${\chi }^{2}$(3, *N* = 62) = 6.31, *p* = 0.097). For the sham condition, however, a significant interaction was demonstrated $({\chi }^{2}$(3, *N* = 62) = 10.02, *p* = 0.018), with an increase in positive scores (value = 3) between baseline and post-training, as revealed by a post-hoc test on the standardized residuals (resid = 3.088, *p* = 0.016; see Fig. 2).

#### Changes in ESM memory bias over time

**Figure 3:**
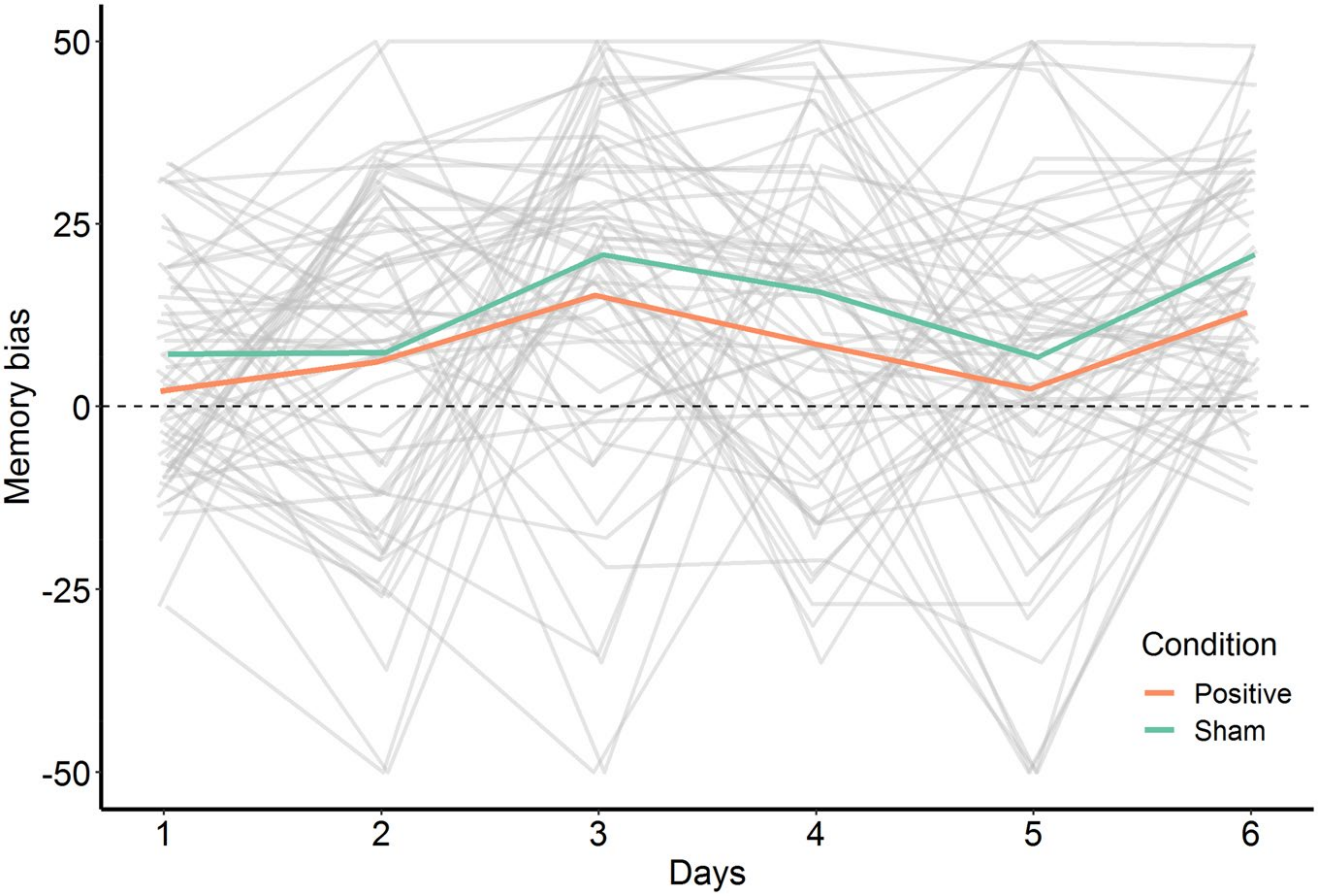
Memory bias scores over the 6 training days. A higher positive value reflects a stronger positive bias. Individual slopes are depicted as grey lines, whereas condition averages are represented as colored lines. The plot depicts a significant main effect of time.

Change of the memory bias score was investigated with a linear mixed-effects model, including a random intercept and slope per participant. No Time × Condition effect was found, (*F*(1, 60) = 0.388, *p* = 0.535). A main effect of Time (*F*(1, 60) = 16.21, *p* < 0.001) revealed a similar change in both conditions, with higher scores on the third day and lower scores on the fifth day (see Fig. 3). This effect was further explored in a supplemental analysis on the effect of time of day on mood (see Supplementary Materials).

#### Changes in mood over time

Effects of the training on changes in mood over time were explored using a linear mixed-effects model. No Time × Condition interaction on positive mood (*F*(1, 59.98) = 0.407, *p* = 0.526) nor negative mood was found (*F*(1, 59.99) = 1.099, *p* = 0.299). A main effect of Time (*F*(1, 59.98) = 9.36, *p* = 0.003) as well as a main effect of Condition was found (*F*(1, 59.995) = 5.089, *p* = 0.028) on positive mood. Post-hoc pairwise comparisons did not show a significant difference in positive mood between the positive and sham condition, irrespective of time. A main effect of condition on negative mood was found (*F*(1, 59.998) = 6.75, *p* = 0.012), suggesting that the sham condition reported a lower negative mood compared to the positive condition ($\Delta$*M* = − 8.38, *p* = 0.036), irrespective of time. In addition, as a supplemental analysis, we explored how changes in mood across the day differed between the two conditions, where moods were significantly more positive in the morning compared to the evening (see Supplementary Materials for more details and results). Lastly, two linear mixed-effects models were performed to explore changes in relaxedness and stress over time. No Time × Condition interactions on relaxedness (*F*(1, 59.98) = 0.29, *p* = 0.592) nor on stress were found (*F*(1, 59.99) = 0.034, *p* = 0.560). However a main effect of Time was found (F(1, 59.98) = 8.16, p = 0.006 and F(1, 59.99) = 6.44, p = 0.014 respectively), suggesting that positive and negative levels of stress changed over time (see supplements for Figure S3).

### Autobiographical memory

**Figure 4:**
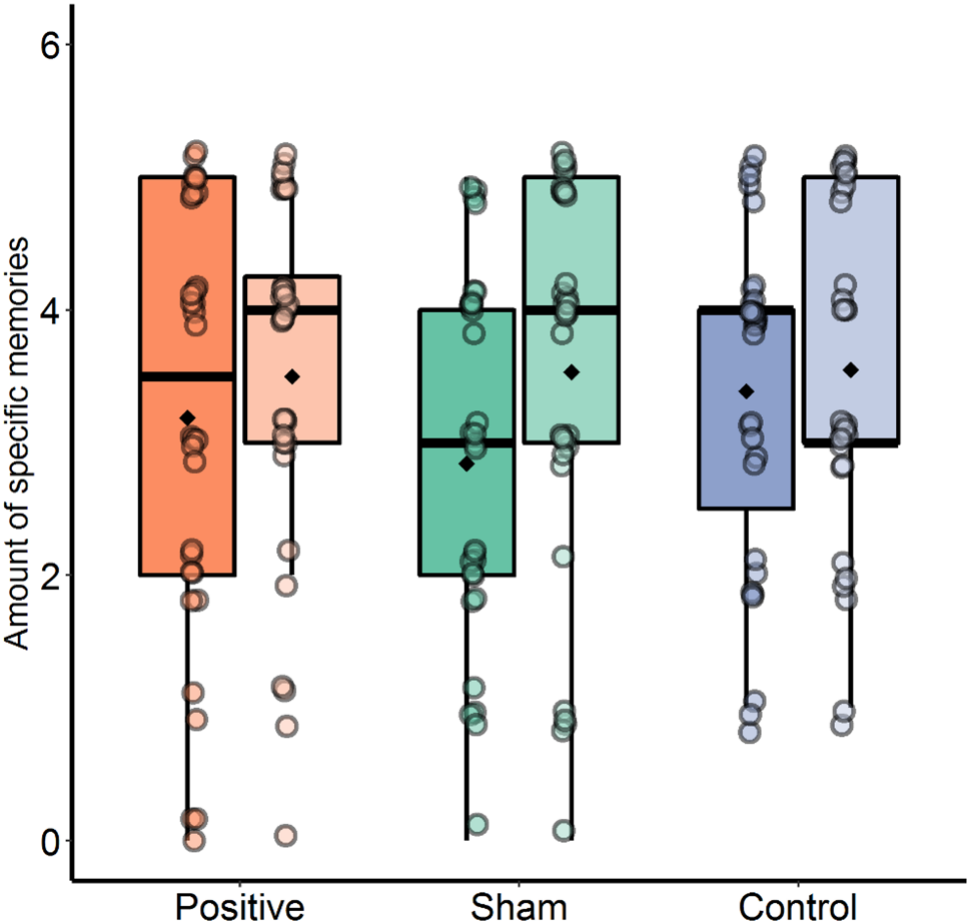
Number of specific autobiographical memories for the three conditions. Each dot represents an individual participant. The dark colors represent the amount of negative memories, whereas the light colors represent the amount of positive memories. The boxes represent the distribution of the amount of specific memories. The box extends from the first to the third quartile. The thicker black line in the middle represents the median, whereas the black diamond represents the mean of each subgroup. The range of values between Q1 and Q3 is also known as an Interquartile range (IQR). The lines that extend from both ends of the box indicate variability outside the first and third quartile.

The average number of specific memories recalled per condition did not significantly differ (*F*(2, 92) = 0.393, *p* = 0.676, ${\eta }^{2}$ = 0.008; see Fig. 4). In addition, the influence of baseline memory bias, as measured by the SRET, on the number of specific memories recalled per training condition was explored. No Pre-memory bias score × Condition interaction on the number of specific memories was found (*p* = 0.935).

Next, we explored if there was a differential effect of the number of recalled specific memories per condition on positive and negative memories. No significant Valence × Condition interaction was found (*F*(2, 92) = 1.425, *p* = 0.246, ${\eta }^{2}$ = 0.006). A main effect of Valence was found (*F*(1, 92) = 8.732, *p* = 0.004, ${\eta }^{2}$ = 0.019), suggesting that irrespective of condition, more specific positive memories (*M* = 3.53) compared to negative memories (*M* = 3.14) were recalled.

### False memory

**Figure 5:**
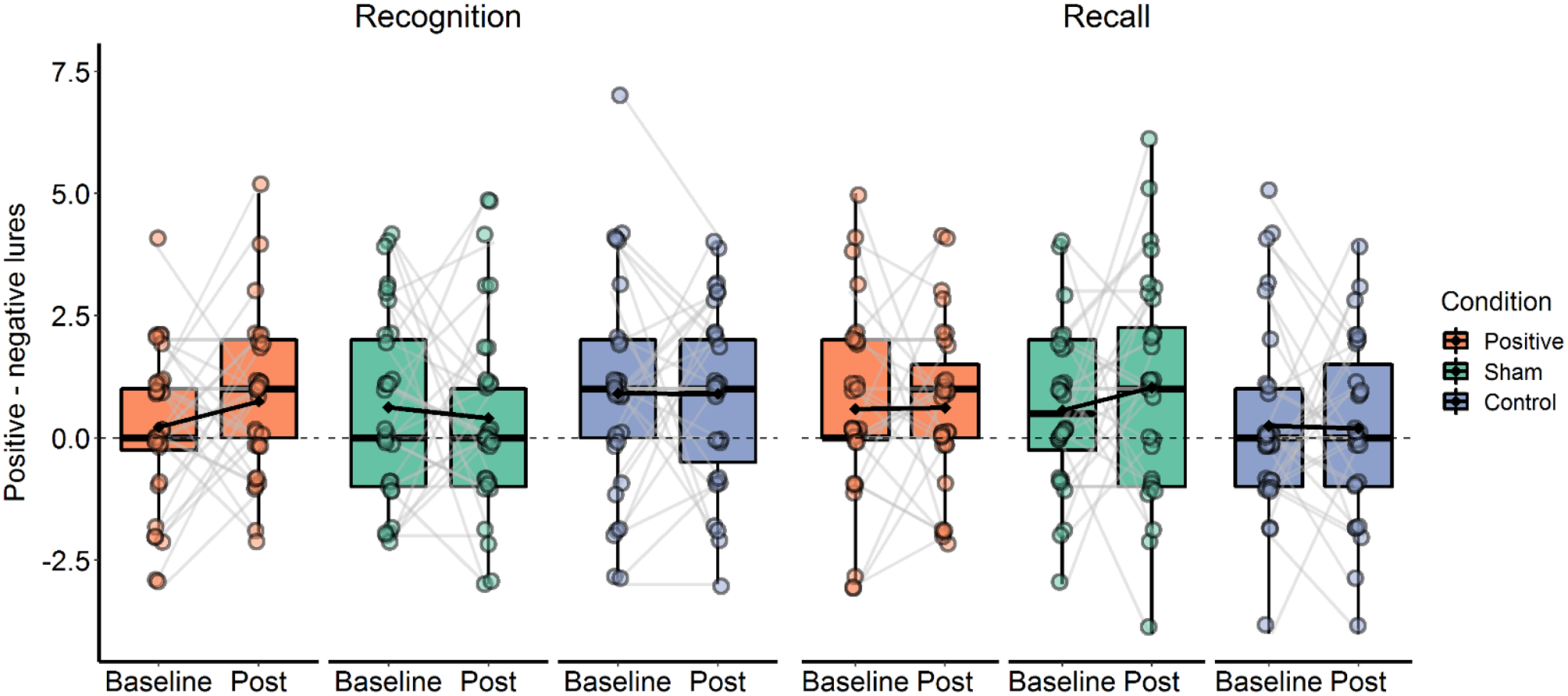
Implicit memory bias was measured by the difference between positive and negative critical lures falsely recognized and recalled over time for the three different conditions. Each dot represents an individual participant. Individual slopes between baseline and post measurement are depicted as grey lines, whereas time point averages are represented as black lines. The boxes represent the distribution of bias. The box extends from the first to the third quartile. The thicker black line in the middle represents the median, whereas the black diamond represents the mean of each subgroup. The range of values between Q1 and Q3 is also known as an Interquartile range (IQR). The lines that extend from both ends of the box indicate variability outside the first and third quartile.

Studied items were recognized better than chance at baseline, (mean d-prime = 0.92, versus chance level of 0), *t*(95) = 17.71, *p* < 0.001), as well as endorsement of critical lures, (mean d-prime critical lures = 1.2, versus chance level of 0), t(95) = 19.84, *p* < 0.001). The number of falsely endorsed critical lures differed per valence at baseline, (*F*(2, 186) = 3.985, *p* = 0.02, ${\eta }^{2}$ = 0.017) with a higher endorsement for positive compared to negative critical lures, *t*(95) = 2.92, *p* = 0.004. The Time × Condition interaction was neither significant for recognition (*F*(2, 92) = 0.767, *p* = 0.467, ${\eta }^{2}$ = 0.007), nor for recall data (*F*(2, 91) = 0.425, *p* = 0.655, ${\eta }^{2}$ = 0.004). No main effect of Time or Condition was found (*p* > 0.5; see Fig. 5).

Next, we explored the influence of baseline memory bias, as measured by the SRET, on the false recognition memory bias difference score between the two sessions for the two training conditions. No Pre-memory bias score × Condition interaction effects were found on false recognition memory bias, (*p* = 0.899) or on the recall data (*p* = 0.055).

### Symptoms

There were no significant Time × Condition interaction effects for the BDI-II (*F*(2, 92) = 0.932, *p* = 0.397, ${\eta }^{2}$ = 0.003), RRS (*F*(2, 92) = 0.434, *p* = 0.649, ${\eta }^{2}$ = 0.0008), PMHS (*F*(2, 92) = 0.2, *p* = 0.819, ${\eta }^{2}$ = 0.0004), and the three subscales of the DASS (depression: *F*(2, 92) = 1.348, *p* = 0.265, ${\eta }^{2}$ = 0.004; anxiety: *F*(2, 92) = 0.569, *p* = 0.568, ${\eta }^{2}$ = 0.002; stress: *F*(2, 92) = 0.967, *p* = 0.384, ${\eta }^{2}$ = 0.004). Significant main effects of time were found for the BDI-II (*F*(1, 92) = 14.815, *p* < 0.001, ${\eta }^{2}$ = 0.023,$\Delta$ M = 2.71), RRS (*F*(1, 92) = 5.391, *p* = 0.022, ${\eta }^{2}$ = 0.005, $\Delta$M = 1.65, and the three DASS subscales (depression: *F*(1, 92) = 4.958, *p* = 0.028, ${\eta }^{2}$ = 0.007, $\Delta$M = 1.41; anxiety: *F*(1, 92) = 7.365, *p* = 0.008, ${\eta }^{2}$ = 0.01, $\Delta$M = 1.39; stress: *F*(1, 92) = 10.973, *p* = 0.001, ${\eta }^{2}$ = 0.022, $\Delta$M = 1.65), indicating an overall decrease in these measures.

In addition, the influence of SRET baseline memory bias on the post-BDI and post-RRS score was examined for the two conditions. No Pre-memory bias score × Condition interaction effects on the BDI score, (*p* = 0.157) nor on the RRS score (*p* = 0.132) were found.

Since both conditions yielded similar increases in positive memory bias, we explored the transfer to depressive symptoms after combining both training conditions and compared this to the no-training (i.e., control) condition. The Time (baseline, post-training)  × Condition (trainings vs. no-training) interaction effect was not significant for the BDI-II (*p* = 0.243), RRS (*p* = 0.621), PMHS (*p* = 0.619), and DASS subscales (depression: *p* = 0.103; anxiety: *p* = 0.815; stress: *p* = 0.472).

### Follow-up after COVID-19 outbreak

Participants repeated the PMHS and the DASS questionnaires online. Conditions did not differ from each other on either the PMHS or DASS scales (all *p *values > 0.2). In addition, there were no Time (post-training, follow-up) × Condition interaction effects (all *p *values > 0.8), indicating that the three conditions did not differentially change in depressive symptoms, anxiety symptoms, stress and positive mental health from post-training to follow-up (see supplements for Figures S4, S5).

**Figure 6:**
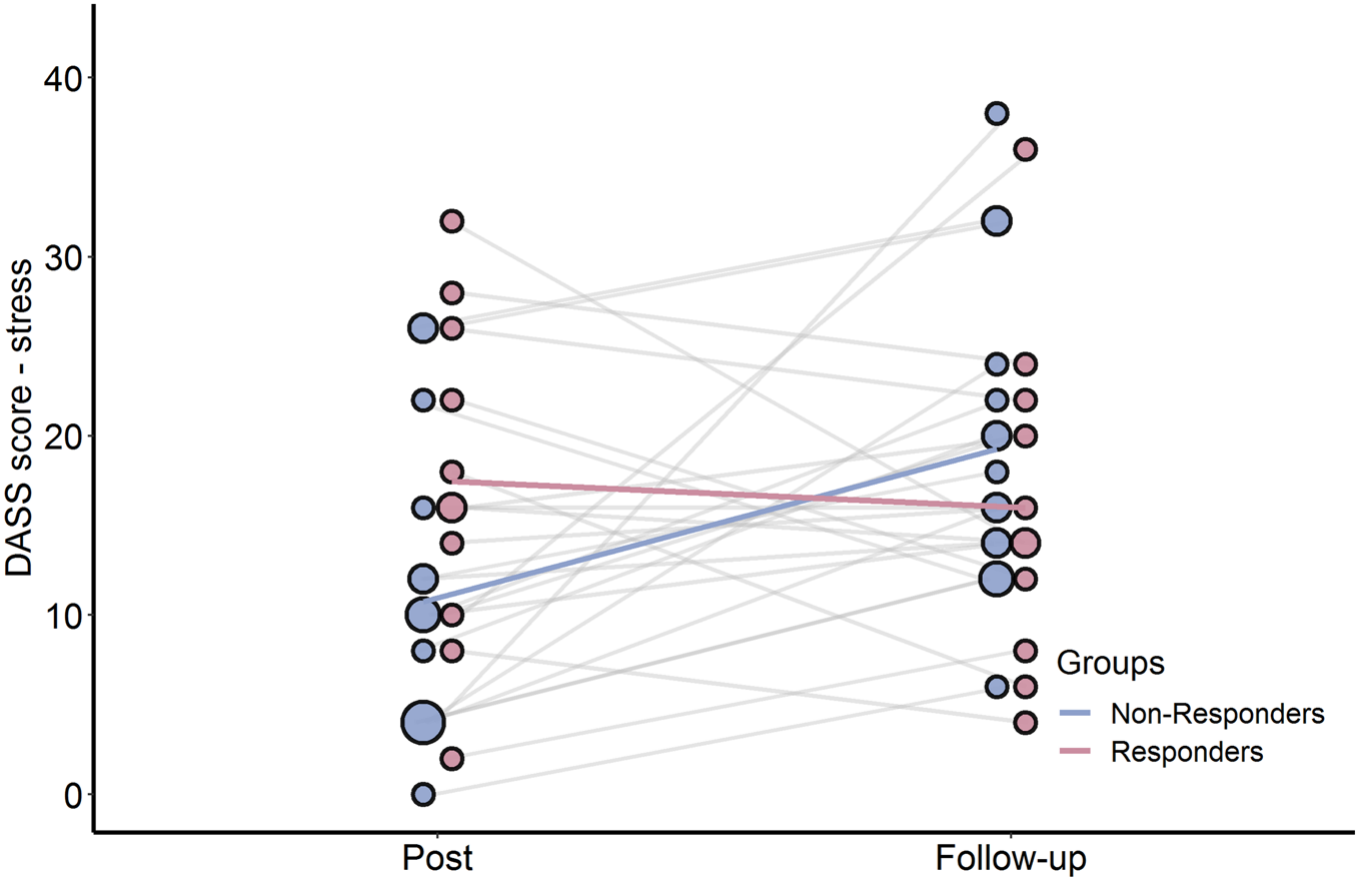
Follow-up. Changes in overall stress scores as measured by the DASS questionnaire at the post measurement and at the follow-up measurement for those that responded most to the training (i.e., responders) to those that responded the least (i.e., non-responders). Dots represent the individual participants, where larger dots represent multiple participants with the same value. Individual slopes between baseline and post measurement are depicted as grey lines, whereas condition averages are represented as colored lines.

Since training effects were similar in both the positive and sham training, we combined the active and sham group and compared those who responded most to the training (i.e., responders) to those who did the least (i.e., non-responders). To identify responders and non-responders, participants were classified as having more change towards a positive memory bias, as measured by the difference score in ESM memory bias between baseline to post-training, compared to the median change (median = 7.67; i.e., responder) versus less change or change towards a negative processing style (i.e., non-responder). Because we could not predict which level of change during these unique times and in this sample should be expected, the median change of the sample was used as benchmark for response. A Time (post-training, follow-up)  × Respond type (responder, non-responder) repeated measures ANCOVA (with Time in weeks between the final session and the followup as a covariate) revealed no significant interactions for the PMHS (*p* = 0.832), DASS depression subscale (*p* = 0.088) and DASS anxiety subscale (*p* = 0.130). However, a significant interaction effect for the DASS stress subscale was observed (*F*(1, 24) = 6.801, *p* = 0.015, ${\eta }^{2}$ = 0.088). A post-hoc pairwise comparisons t-test showed that the non-responder group showed a significant increase in stress levels between post measurement (Δ*M* = 8.4, *p* = 0.007), whereas the responder group remained stable over time (Δ*M* = − 1.5, *p* = 0.712; see Fig. 6). Lastly, we wanted to provide context to the typical trend of changes in stress, depression, and anxiety during the follow-up period by comparing the responder and non-responder groups to those that did not receive any training at all. Hence, we repeated the ANOVAs comparing the responder (n = 11), non-responder (n = 16) and the no-training (n = 7) groups on the DASS subscales and PMHS during the follow up time point. However, no group differences were found for the DASS stress subscale (*F*(2, 31) = 0.653, *p* = 0.528, *η*^2^ = 0.04), the DASS depression subscale (*F*(2, 31) = 0.025, *p* = 0.975, *η*^2^ = 0.002), the DASS anxiety subscale (*F*(2, 31) = 0.522, *p* = 0.598, *η*^2^ = 0.033) nor the PMHS scores (*F*(2, 31) = 0.205, *p* = 0.816, *η*^2^ = 0.013).

## Discussion

In the current study, we aimed to modulate memory bias in dysphoric individuals using a novel smartphone-based app that allowed for an ecologically valid training in the participant’s own environment, which could bolster transfer of training effects to daily life cognitive processing. We compared the effects of positive training, sham training and no-training on memory bias and depressive symptoms.

The results did not show a differential effect of training type (positive compared to sham training), but show an overall increase in positivity bias, as well as overall increase in positive mood over time. Thus, both trainings may be equally effective. The sham training’s effectiveness is further reflected by the significant increase in the actual total number of positive recalled memories between baseline and post-training. The sham training was designed to be a neutral alternative to the positive training, as it was expected that participants would show a general neutral attitude towards their environment across the day (e.g., sitting at home or being at work) and repeated reflection would thereby minimally influence memory bias. However, it could be that such repeated contemplation on one’s current environment leads to an indirect training of attentional focus or control. Re-focusing attention to the contextual environment has been shown to decrease maladaptive rumination in mindfulness training^40^, in which participants are instructed to focus their attention on the “here and now”, Mindfulness has been linked to the enhancement of attentional control^41,42^, which ultimately strengthens the ability to disengage from ruminative thoughts. While it generally targets a broader field of behavioral processes, including acceptance, releasement of judgement and cognitive defusion^43,44^, similarities with our sham condition are apparent. In addition, contrary to expectation, participants rated their environmental context as pleasant, instead of neutral, and unexpectedly rated their recalled events as more positive than the positive condition. Possibly, such context reflection cues lead to an overall—perhaps dormant—appreciation of the environment and should be integrated in future trainings. Along these lines, the earlier reported large ineffectiveness of general CBM compared to sham training^16^, might in fact be due to an unintended effectiveness of the sham condition. Also in other CBM paradigms, the sham condition has been found to increase cognitive flexibility^45^. The finding that the sham condition yielded more positive memory than the CBM-Memory condition could also be explained by a difference in ‘time frame’ of recall, as the active condition trains retrospective bias, while the sham condition focuses on the here and now possibly also tapping into processes such as ‘savoring’ and ‘focused attention effects.

Alternatively, our null results on the differential effect of training type may reflect an ineffectiveness of either training. Equivalence tests can determine whether a true effect is close enough to zero that it can be considered practically meaningless—smallest effect of interest (SESOI^46^) and can hence be informative if a replication (in an adequately powered sample) provides similar null results. In line with our findings, previous studies have uncovered certain “stubbornness” in modifying bias, where healthy individuals seem resilient against developing a negative memory bias^28^, or only dysphoric individuals with an initial positive processing style show positive training effects^26^. In our sample, the observed increase in positive bias over time across both conditions may reflect a regression to the mean given the pre-selection on depressive symptoms. However, a large portion of the included participants already showed decreased depressive symptoms at baseline compared to the pre-screening, demonstrating the variability of this measurement. As such, even though our sample represents a vulnerable group of individuals, their depressive symptoms may not be stable, which could thereby be reflected by the increase in positive memory bias scores over time, independent of training. Future studies should include an identical memory bias measurement in a control group without training to exclude this speculation and to specifically map natural memory bias change over time.

Next, we examined possible transfer effects of the training compared to no training. No transfer effects were found for any of the training conditions on autobiographical memory specificity, implicit memory bias or depressive symptoms. Possibly, even though the current training was twice the length of the previous pilot study^28^, the 6-day training may still have been of insufficient dosage to not just alter persistent biases, but to also see effects on related, but distinct symptoms. Additionally, training effects might show a delay, which is often not captured in CBM studies including no or only short-term follow-ups^47^. A promising clue regarding delayed training effect comes from our follow-up results, although we did not find differences between the positive and sham condition even at follow-up. The follow-up measurement took place amid the initial COVID-19 outbreak—a generally stressful period. Using this data, we could explore possible long-term effects of training on resilient responses to stress. Non-responders showed a significant increase in self-reported stress levels between post-training and follow-up measurement, whereas responders showed no significant change. Ultimately, it seems that training success may only be observed after a longer period of time, although it remains unclear if this is due to continuation of training or a delayed response to the training itself. It is important to note that there was still no difference between the training conditions; the positive and sham condition performed similarly. In the follow-up data, we find that responders to either the active or sham training seem more resilient during the COVID pandemic, and both conditions yielded memory bias relief. However, because the intervention target (focusing on positive memories versus the current context) differed between condition, we cannot ascribe this more positive bias to a specific training condition. Relatedly, these results may indicate that individuals who adapt more easily to environmental demands (i.e., cognitive training, changing environments) may be more are resilient to stress. Overall, the results may suggest that either training type can increase stress resilience, or alternatively, more resilient people are more susceptible to training effects. Indeed, a recent large scale cross-sectional study demonstrated that adolescents who showed greater resilience, showed more positive memory biases prospectively^48^.

While assessing the effectiveness of modifying separate cognitive biases (e.g., attention, interpretation, memory) is extremely valuable for the investigation of their distinct roles in the development and maintenance of depression and other psychiatric disorders, future studies should ultimately consider developing trainings that target multiple cognitive biases at once. Though potential positive outcomes may thereby lack specificity for the underlying cognitive mechanisms, they would be invaluable for future treatment development. Recent work has tried to uncover the complex interactions between distinct biases, formally coined as the combined cognitive bias hypothesis^49,50^, but has not yet been translated to appropriate training protocols for potential future treatments. At home training approaches, as used in the current study, seem to be promising, practical and inexpensive add-ons to cognitive behavioral therapies^51^ or ways to boost early effects of antidepressants^52^, although potential limitations such as limited control over degree of active engagement in the training should be considered. A limitation is that the study sample included a majority of females and was conducted in a student population, which limited the generalizability of the findings. Although the COVID-19 pandemic can be perceived as a ‘global stressor’ impacting individuals in their daily life^53^, we did not control for other potential confounding variables during the follow-up period and consider this a limitation to the current findings. Also, the smartphone-based memory bias measure was not available for the no-training condition, because they did not receive a smartphone and were tasked as minimally as needed. Hence, we are unable to illustrate the natural change in memory bias across the intervention period and conduct comparisons between the positive condition, sham condition and no-training condition. We consider this an important limitation to the interpretation of the current findings. Inclusion of the no-training condition does fortunately allow us to show naturalistic change in distress and depressive symptoms, serving as a benchmark in the comparison with the two training conditions.

Overall, while we could not see any specific short-term effects of the positive compared to sham training on memory bias or depressive symptoms, we did find a first indication of potential long-term effects of successful bias change (irrespective of training type). This, however, should be weighed against the limitation that the current study design does not allow for investigations of natural change in memory bias across time. Future studies would greatly benefit from designing a truly non-emotional sham condition, as well as including the possibility to compare memory bias change to a no-training group and to assess long-term effects in their study design.

## Supplementary Information

Supplementary Information.

## Data Availability

The data that support the findings of this study are available on request from the corresponding author. The data are not publicly available due to privacy restrictions since the data include descriptions of locations and events unique to the participant.
