# Supplementary material for: The effects of daily autobiographical memory training on memory bias, mood and stress resilience in dysphoric individuals
Source: Sci Rep. 2022 Dec 3;12:20873. doi: 10.1038/s41598-022-25379-9 (PMC9719463; doi:10.1038/s41598-022-25379-9)
Supplement: Supplementary file 1 — Supplementary Information. [file 41598_2022_25379_MOESM1_ESM.docx]

# **Supplemental materials**

The Effects of Daily Autobiographical Memory Training on Memory Bias, Mood and Stress Resilience in Dysphoric Individuals

Leonore Bovy^1^, Nessa Ikani^2,3^, Livia N.M. van de Kraats^2^, Martin Dresler^1^, Indira Tendolkar^1,2^ and Janna N. Vrijsen^2,4*^

^1^ Donders Institute of Cognition and Behaviour, Radboud University Medical Center, Centre for Cognitive Neuroimaging, Nijmegen, The Netherlands

^2^ Department of Psychiatry, Donders Institute for Brain, Cognition and Behaviour, Radboud University Medical Center, Nijmegen, The Netherlands

^3^ Behavioural Science Institute, Radboud University, Nijmegen, The Netherlands

^4^ Depression Expertise Centre, Pro Persona Mental Health Care, Nijmegen, The Netherlands

# **Methods**

**Figure S1**

*Overview of the experience sampling method (ESM) design*

| **Prompt** | **Timeslot** | **Day 1** | **Day 2** | **Day 3** | **Day 4** | **Day 5** | **Day 6** |
| --- | --- | --- | --- | --- | --- | --- | --- |
| **1** | 08:00–09:45 | Sleep quality &  Mood | Sleep quality &  Mood &  **Training** | Sleep quality &  Mood | Sleep quality &  Mood &  **Training** | Sleep quality &  Mood &  **Memory bias** | Sleep quality &  Mood |
| **2** | 09:45–11:30 | Mood &  **Memory bias** | Mood &  **Training** | Mood &  **Training** | Mood &  **Training** | Mood &  **Training** | Mood &  **Training** |
| **3** | 11:30–13:15 | Mood &  **Memory bias** | Mood | Mood &  **Training** | Mood &  **Memory bias** | Mood &  **Training** | Mood |
| **4** | 13:15–15:00 | Mood &  **Memory bias** | Mood &  **Training** | Mood &  **Training** | Mood &  **Training** | Mood | Mood &  **Training** |
| **5** | 15:00–16:45 | Mood &  **Training** | Mood &  **Memory** **bias** | Mood | Mood &  **Training** | Mood &  **Training** | Mood &  **Training** |
| **6** | 16:45–18:30 | Mood &  **Training** | Mood &  **Training** | Mood &  **Training** | Mood | Mood &  **Training** | Mood &  **Memory bias** |
| **7** | 18:30–20:15 | Mood | Mood &  **Training** | Mood &  **Memory bias** | Mood &  **Training** | Mood &  **Training** | Mood &  **Memory bias** |
| **8** | 20:15–22:00 | Mood &  **Training** &  End of day | Mood &  End of day | Mood &  **Training** &  End of day | Mood &  End of day | Mood &  End of day | Mood &  **Memory bias** &  End of day |
| *Note.* Overview of the experience sampling method (ESM) design. Training lasted for 6 days and included 8 prompts per day. The first prompt was started by the participant via the smartphone app. All subsequent prompts were sent randomly within specific timeslots across the day. Prompts were either positive or sham training prompts (depending on condition; in orange), a memory bias assessment (in green), a mood assessment, a sleep quality assessment or an assessment at the end of the day probing anhedonia, sense of mastery and amount of physical exercise. The sleep and end of the day assessments served as pilot data for a subsequent study and are not part of the current research question*.* | | | | | | | |

## **Self-referential encoding task**

In the self-referential encoding task (SRET^1^), twelve positive and twelve negative adjectives were presented on a computer screen, in a fixed randomized order with no more than two subsequent words of the same valence. Participants were instructed to indicate if the word describes them or not(endorsement). Next, subjects performed a paper-based distraction task (2 minutes), followed by a surprise memory test, in which they had to recall as many words as possible from the SRET. Close synonyms of task words and typos were counted as correct - checked by two independent raters. In line with an earlier study ^2^, the first and last two words were omitted from the analysis to reduce primacy and recency. The amount of positive endorsed-as-self-descriptive and subsequently recalled words were divided by the total number of endorsed and recalled words per participant, to calculate a positive recall bias score ^3^. The bias score ranged between 0 and 1, where higher scores represent more positive memory bias.

## **Autobiographical memory task**

The autobiographical memory task (AMT; ^4^ assessed the long-term training transfer effects to autobiographical memory. Subjects were instructed to recall a personal memory (a specific event, lasting no longer than one day) related to positive and negative cue words (n=5 per valence, therefore n=10 total) and describe it. All events were scored by an independent rater on whether they were specific, general (categoric or extended) or other (no response, no memory, same memory as recalled before); the variable of interest was the number of specific memories recalled^3^.

## **False memory task**

An emotional version of the Deese-Roediger-McDermott (DRM) false memory task ^5^ was used to probe implicit memory bias. Lists of emotional (positive, negative and neutral) semantically related words were constructed in both Dutch and German (see ^6^ for more information on the task construction). In short, each list consisted of 10 related words, all corresponding to an unpresented critical lure. Each word was presented twice (first in order of strongest to weakest association with the critical lure, then in semi-random order) on a computer screen for 750ms. To ensure participants were paying attention two questions followed each list, regarding the subject’s current mood and meta-memory (number estimate of words remembered); responses were measured on a continuous scale.

Immediately afterwards, participants were required to freely recall as many words as possible. Typos or pluralization of correct words were marked by two independent raters (one Dutch and one German) and accepted as correct responses. In addition, alternatives of the pre-determined critical lures - fitting to the list of related words - were also determined by the independent raters and included in the analysis as critical lures.

During recognition, three old, three new, and each critical lure per list were semi-randomly presented on the computer screen. Participants had to indicate via button press how sure they were the word was old or new (sure old, somewhat sure old, and unsure old and sure new, somewhat sure new, and unsure new) within 3s. The hit, false alarm, and critical-lure rates were calculated per valence category (negative, neutral, positive) per participant. In addition, a discrimination ability index (d-prime) was calculated as follows: d′=z(H)−z(FA). A higher value indicates that a subject had greater ability to discriminate between studied and unstudied items. In addition, a d-prime value on the sensitivity to falsely recognizing critical lures was calculated as follows: d′critical_intrusions=z(FA_critical_intrusion)−z(FA). Here, higher values suggest a higher propensity to falsely recognize lures. If the hit rate or (critical) false alarm rate was 1, it was replaced with 1 - 1/(2N), where N represents the number of items for that category (e.g. 300 for all old items). If the rate was 0, it was replaced by 1/(2N).

## **Questionnaires on mental state**

The Beck Depression Inventory assesses depressive symptom severity and consists of 21 items that can receive a score from 0 to 3. The total sum score represents a minimal (0-13), mild (14-19), moderate (20-28) or severe (>28) depression ^7^. At baseline, the standard instruction was used assessing symptoms across the last *two* weeks; the instruction was adapted to represent symptoms across the last week during the post measure. The Ruminative Response Scale (RRS; ^8^ assesses ruminative trait responses to depressed mood, using 26 items scored between 1 and 4 each. Higher scores reflect more rumination. The Positive Mental Health Scale (PMHS; ^9^ assesses positive mental health, using 9 Likert-type items, scored between 1 and 4 each. The higher the score, the more positive health. The Depression Anxiety Stress Scales (DASS; ^10^ assesses both depression, anxiety, and stress levels using 21 items, scored between 0 and 3. Three subscales can be created from this questionnaire; a depression, anxiety and stress subscale.

# **Results**

Several supplemental control analyses were performed to support the main analysis on memory bias.

## **Memory bias**

##### Training effects on memory bias: on consistently dysphoric sample ($\geq$ 14 BDI)

As a post-hoc exploratory analysis, we wanted to perform the same analysis in those participants that scored $\geq$ 14 on the BDI-II scale at baseline, and were hence consistently dysphoric. In the active group, 20 participants and in the control 23 participants scored more than 13 on the BDI-II scale at baseline. No significant interaction between time and condition was found on the average memory bias score (*F*(1, 41) = 1.11, *p* = .298, $\eta^{2}$ = .01). A main effect of time was found, (*F*(1, 41) = 12.89, *p* <.001, $\eta^{2}$ = .102), indicating an overall increase towards positive bias over time for both conditions ($\Delta$M = 10.28). No main effect of group was found (*p* = .150).

##### Training effects on memory bias: moderation effect of prior positive bias

Next, we checked if an a-priori positive bias (as measured by the SRET task) moderated the training effect. No interaction between SRET bias score and group on the difference score (i.e. average of last three days minus the average of first three days) of the ESM memory bias was found, (*F*(1, 58)=1.034, *p*=.313, $\eta^{2}$=.008).

##### Changes in mood across the day over time

Next, we explored how changes in mood across the day differed between the two conditions. Here, three prompts sent between 08:00 and 13:15 were classified as “morning” prompts, two prompts sent between 13:15 and 16:45 were classified as “afternoon” prompts and three prompts sent between 16:45 and 22:00 were classified as “evening” prompts. No three-way interaction between condition, time and time of day were found on positive mood, (*F*(2, 2805.13)=2.67, *p*=.069), nor on negative mood, (*F*(2, 2804.08)=2.31, *p*=.099). However, besides main effects of time and condition on positive and negative mood (time: *p* <.001, *p*=.007, condition: *p*=.037, *p*=.01 resp.), a significant main effect of time of day was found on positive (*F*(2, 2805.15)=8.52, *p* < .001) and negative mood (*F*(2, 2804.09)=3.56, *p*=.028). These results show that general mood was more positive in the evening compared to the afternoon ($\Delta$M=5.27) or compared to the morning ($\Delta$M=6.39), and less negative in the evening compared to the afternoon ($\Delta$M=-3.6) or compared to the morning ($\Delta$M=-2.94) in both conditions (see Figure S2). These findings may explain the fluctuating scores over time in the memory bias reported in the previous results (see Figure 3 in main text). At day 3, the memory bias score was assessed in the evening whereas the bias score was assessed in the morning at day 5 (Figure S1). Given that mood seemed to be generally higher in the evening, mood may explain the changes over time in the memory bias.

*Brooding subscale of the Ruminative Response Scale*

Different cognitive responses to stressful events play a powerful role in the onset, maintenance and amount of depressive symptoms. In particular brooding has been recognized as playing a central role in this^11^. Therefore, we looked into group differences on the brooding subscale of the RRS between our active, sham and no-training groups during the post-training session as an exploratory analysis. No group differences were found, *F*(2, 92)=0,398, *p*=.673, *η*^2^=.009.

**Figure S2**

*Positive and negative mood changes across the day over the 6 training days between conditions*


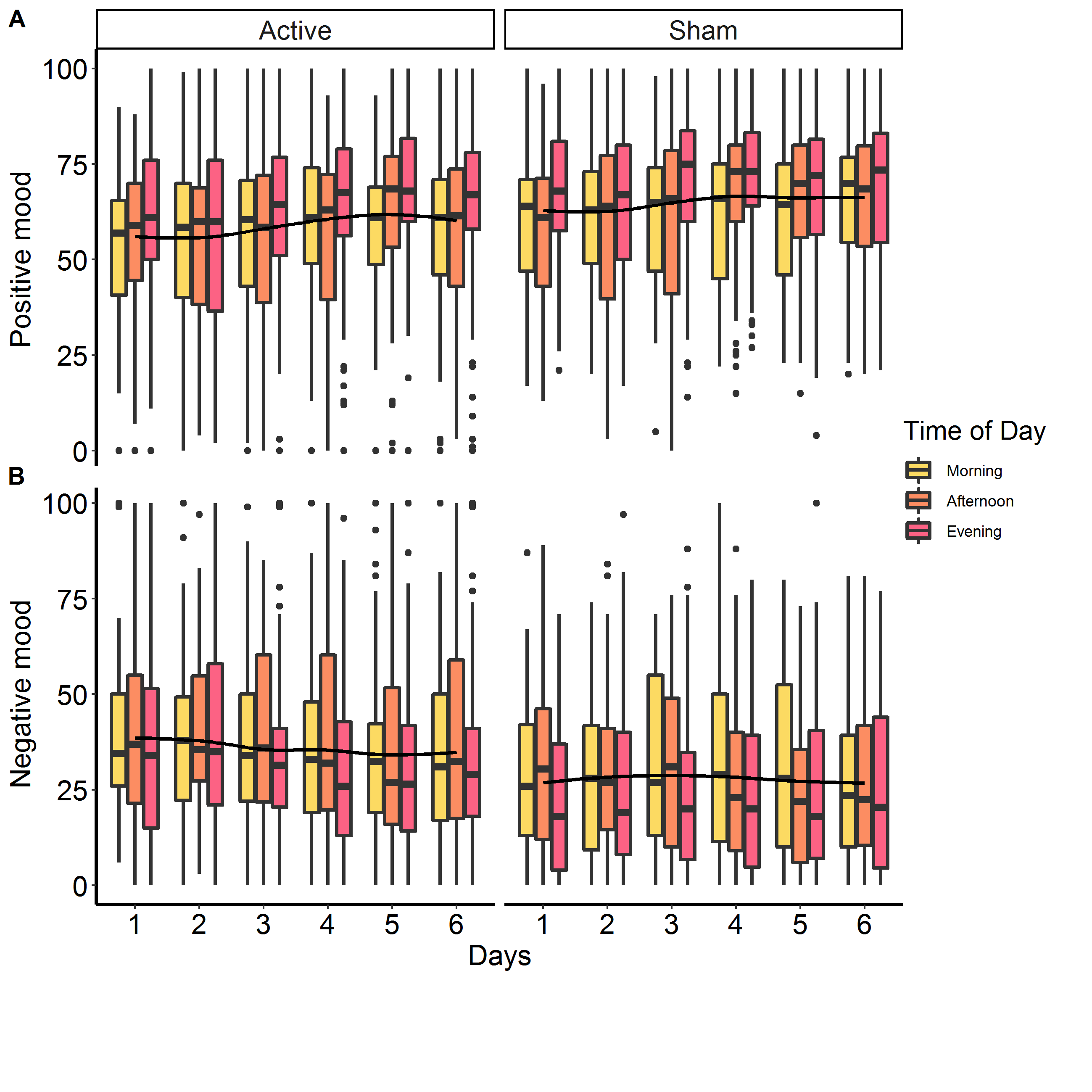


Note. Lower and upper hinges correspond to the first and third quartiles (the 25th and 75th percentiles). The whiskers extend from the hinge to the largest value no further than 1.5 * inter-quartile range from the hinge. Data beyond the end of the whiskers are considers outliers and are plotted individually. The plot depicts a significant main effect of time as well as a main effect of time of day for both positive (**A**) and negative (**B**) mood.

**Figure S3**

Levels of self-reported relaxedness and stress across the day over the 6 training days between conditions


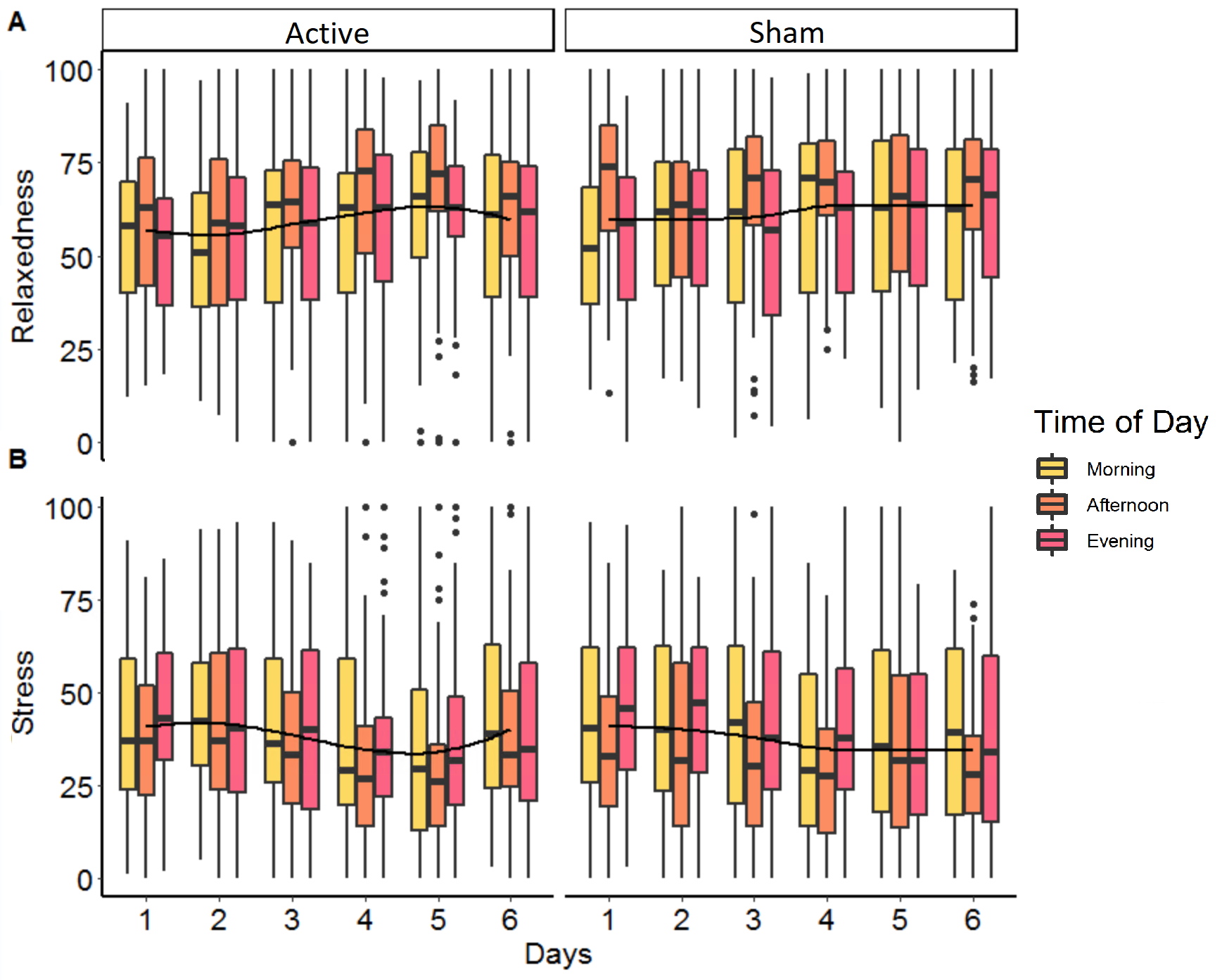


Note. Lower and upper hinges correspond to the first and third quartiles (the 25th and 75th percentiles). The whiskers extend from the hinge to the largest value no further than 1.5 * inter-quartile range from the hinge. Data beyond the end of the whiskers are considers outliers and are plotted individually. The plot depicts a significant main effect of time as well as a main effect of time of day for both relaxedness (A) and stress (B).

**Figure S4**

*PMHS scores before training, after training and at follow-up after COVID-19 outbreak.*

**
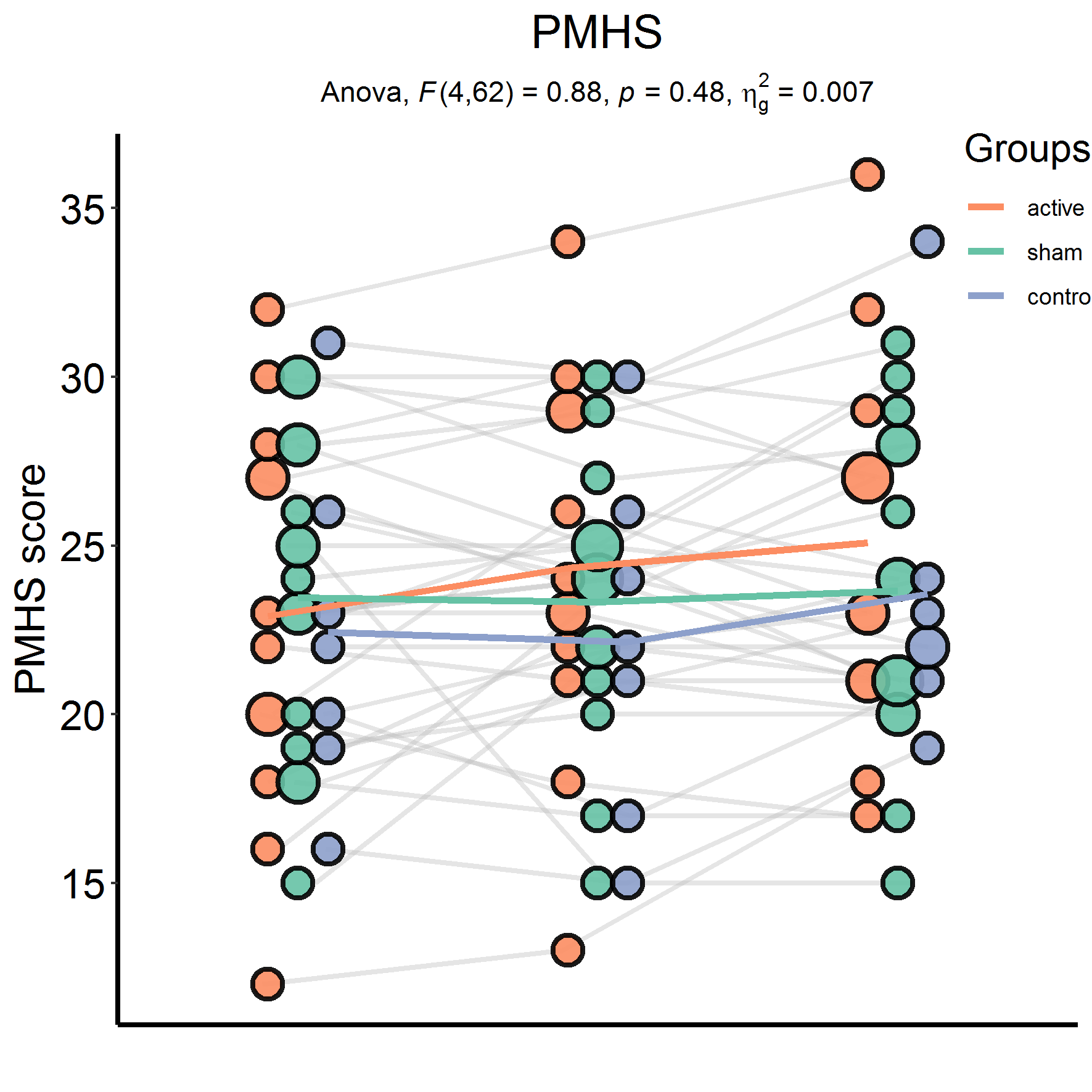
**

**Figure S5**

*DASS subscale scores before training, after training and at follow-up after COVID-19 outbreak.*

**
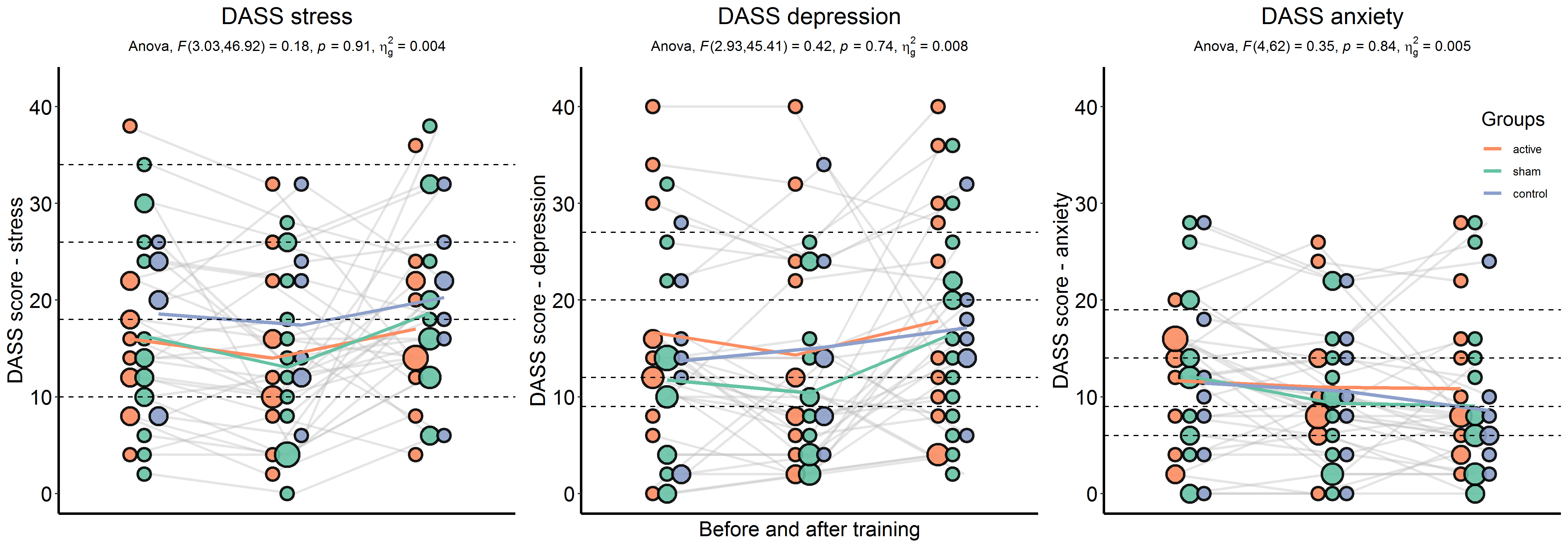
**

**
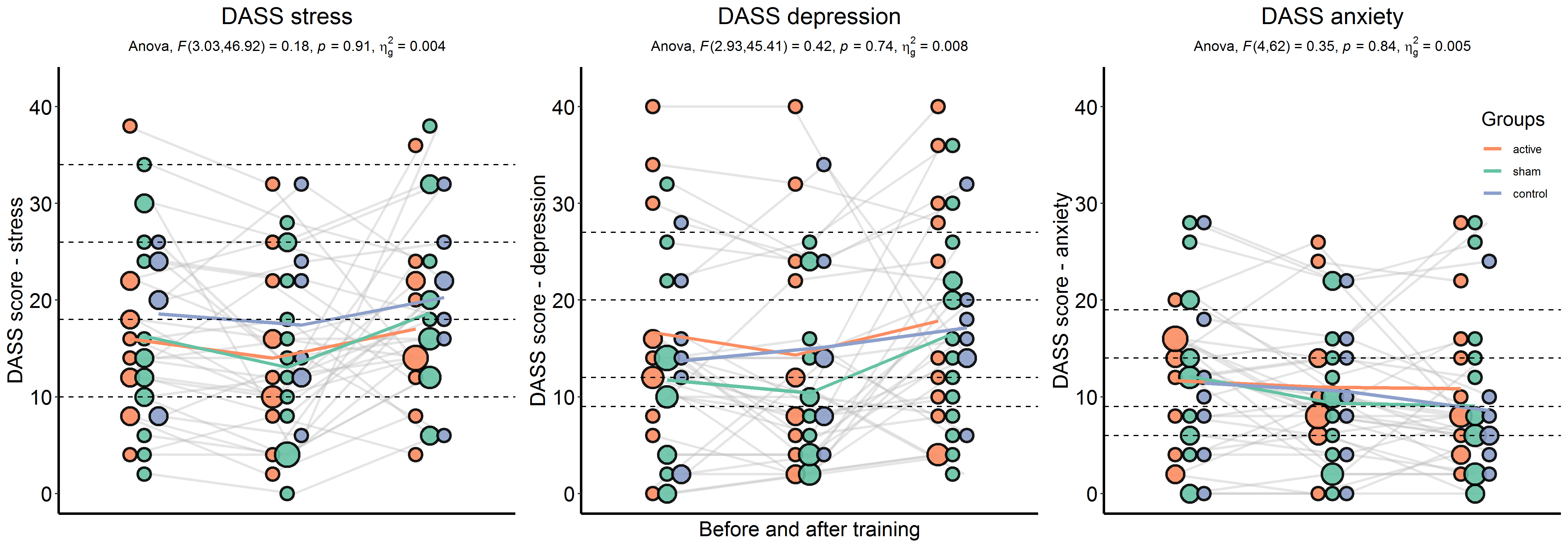
**

**
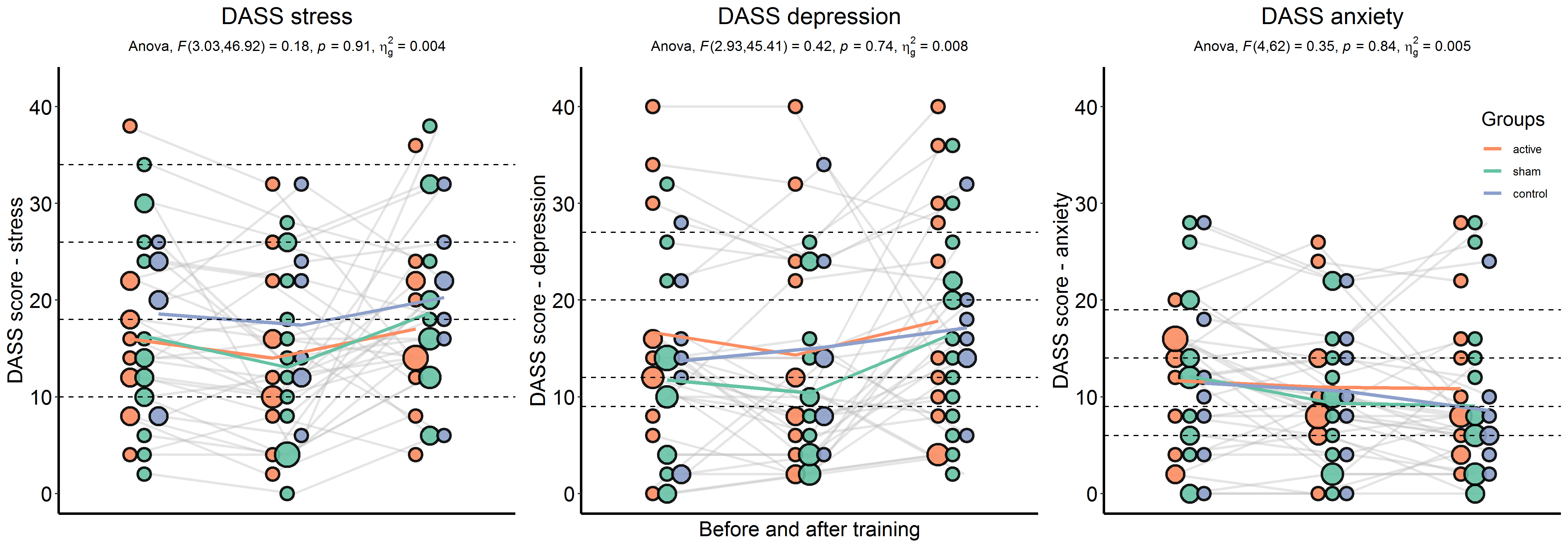
**

# **References**

1. Derry, P. A. & Kuiper, N. A. Schematic processing and self-reference in clinical depression. *J Abnorm Psychol* **90**, 286–297 (1981).

2. Vrijsen, J. N. *et al.* Effect of cognitive bias modification-memory on depressive symptoms and autobiographical memory bias: two independent studies in high-ruminating and dysphoric samples. *Cognition and Emotion* **33**, 288–304 (2019).

3. Gotlib, I. H., Krasnoperova, E., Neubauer Yue, D. & Joormann, J. Attentional Biases for Negative Interpersonal Stimuli in Clinical Depression. *Journal of Abnormal Psychology* **113**, 127–135 (2004).

4. Crovitz, H. F. & Schiffman, H. Frequency of episodic memories as a function of their age. *Bull. Psychon. Soc.* **4**, 517–518 (1974).

5. Roediger, H. L. & McDermott, K. B. Creating False Memories: Remembering Words Not Presented in Lists. *Journal of experimental psychology. Learning, memory, and cognition* **21**, 803–814 (1995).

6. Bovy, L. *et al.* Transcranial Magnetic Stimulation of the Medial Prefrontal Cortex Decreases Emotional Memory Schemas. *Cereb Cortex* **30**, 3608–3616 (2020).

7. Beck, A. T., Steer, R. A. & Brown, G. K. Beck depression inventory-II. *San Antonio* **78**, 490–498 (1996).

8. Treynor, W., Gonzalez, R. & Nolen-Hoeksema, S. Rumination Reconsidered: A Psychometric Analysis. *Cognitive Therapy and Research* **27**, 247–259 (2003).

9. Lukat, J., Margraf, J., Lutz, R., van der Veld, W. M. & Becker, E. S. Psychometric properties of the Positive Mental Health Scale (PMH-scale). *BMC Psychology* **4**, 8 (2016).

10. Lovibond, P. F. & Lovibond, S. H. The structure of negative emotional states: Comparison of the Depression Anxiety Stress Scales (DASS) with the Beck Depression and Anxiety Inventories. *Behaviour Research and Therapy* **33**, 335–343 (1995).

11. Marroquín, B. M., Fontes, M., Scilletta, A. & Miranda, R. Ruminative subtypes and coping responses: Active and passive pathways to depressive symptoms. *Cognition and Emotion* **24**, 1446–1455 (2010).
